# A Question of Sanatorium Control

**Published:** 1913-06-28

**Authors:** 


### A QUESTION OF SANATORIUM CONTROL.

A recent paragraph under the above heading has produced a letter, given elsewhere, from one of those rather rare officials, a sanatorium steward, suggesting that it is not necessary for the medical superintendent to have control of the outdoor servants before "graduated labour" is introduced among the patients. There are several things to be said on this subject, with some of which our readers will be already familiar. We have previously pointed out that the sanatoriums which first of all made a name were those entirely directed by one man—Bodington, Brehmer, Dettweiler, Walther, Trudeau. These early institutions, although some of them of considerable size, were in the main private ones. But since then Dr. Paterson has at any rate completely proved that the system, given a rather exceptional person, is the best of all for public sanatoriums too. We have also endeavoured to show to what this state of affairs traces. Hospital patients are mostly very sick people, and, in consequence, very manageable. Women have the immediate charge of them; and the feminine inspiration of the nursing system unconsciously provides additional helps to discipline by relying on the impressive effects of two things—namely, great care for appearance and frequent religious services. The contrast, to continue travelling over old ground, was then made of sanatorium patients. They are soon highly convalescent, and even plethoric and concupiscent. They have the *spes phthisica.* On the auto-inoculation theory and because it would make them cough, it is inadvisable for them to be always singing to a harmonium. Yet these rather vigorous people require their way of life mapped out for them more minutely than for school-children, their exercise regulated to a foot-pound, while order and morality must be firmly maintained. Hence the need for another kind of prestige, not that emanating from wards, but that of your Walthers and your Patersons. This will obviously be helped, and their organising power given a chance, by their having control of the outdoor servants.

All other points generic to this discussion seem to us to come into view from a single, but vital, aspect of the subject—namely, the economic one. Consider the modern anti-tuberculosis programme. As regards institutions, there are the sanatorium, the dispensary, the home for advanced cases, the farm colony; all of course with paid staffs. Then the consumptive's dependants must be maintained during his absence, while if he stops at home he needs milk and cod-liver oil and tuberculin. It really matters nothing talking about the Insurance Act. The ultimate, inevitable fact of the situation is that every sick man or woman who is not working is a burden upon those who are working, that is, upon the rest of the community, and that of such burdens the consumptive is the one who has to be carried furthest. Many Insurance Committees are now expending all they dare of available funds in keeping a handful of patients at some sanatorium. So that when one hears the expression—"before graduated labour is introduced," one can only boggle at it. "Graduated labour" is very probably of no therapeutic value, but economically it is a discovery of prime importance. What a good thing to let the invalid himself help a little, since it is certain he can do so without any physical harm and with distinct moral gain! How all this fits in with the picture outlined above—a modest establishment without meticulous spick and spanness, with a small staff of subordinates, the help of patients utilised to the full, the medical resident a highly practical administrator as well as a competent clinician. The need of sanatorium administration is most emphatically for efficiency and economy before appearance.

If sanatoria grow to the size of mental hospitals, then a steward is a necessity and must be given adequate administrative control. But probably this will happen very seldom. Although, perhaps, there are even more people with technically unsound lungs than of unsound mind, yet clinically, tuberculous patients fit for a sanatorium are far fewer. The county sanatoria will be much smaller affairs than the county mental hospitals, and in the direction of them there will not be much question as to direct control of outdoor servants by the medical resident. Already the Winsley Sanatorium is one of a small minority; and it has had four medical superintendents in the last six years.

